# A Technical Note on Alternating Laminectomies Plus Folding Cystectomy: A Novel Technique for Spinal Arachnoid Cyst

**DOI:** 10.7759/cureus.54978

**Published:** 2024-02-26

**Authors:** Obet Jair Canela-Calderon, Sergio Ramírez-Aragón, Jorge Del Pino-Camposeco, Eliezer Villanueva-Castro, Juan Antonio Ponce-Gómez, Juan Nicasio Arriada-Mendicoa

**Affiliations:** 1 Department of Neurosurgery, Instituto Nacional de Neurología y Neurocirugía Manuel Velasco Suárez, Mexico City, MEX

**Keywords:** spine surgery, giant epidural cyst, laminoplasty, laminectomy, spinal arachnoid cyst

## Abstract

We report the case of a 33-year-old male patient with no past medical history presenting to our tertiary referral center with progressive (two years) deficit of lower limb motor impairment (2/5 Medical Research Council [MRC] scale) and sensory impairment. T2- and T1-weighted MRI images clarified the nature of the cyst from T3 to T8. In our case, surgical management was warranted to relieve tension over the spinal cord, thus improving symptoms. Two multilevel laminectomies were performed, one centered on the proximal pole and the other on the distal pole; subsequently, the epidural cyst was gradually folded until it was totally extracted without complications. In the present study, we discuss a technique of extended spinal compressive arachnoid cyst. To the best of our knowledge, this technique has not been previously described in the existing body of literature. Here, we present a case of a successful procedure that seems both efficient and safe. Further study will be required to confirm its safety and efficacy.

## Introduction

Spinal arachnoid cyst is a rare entity, accounting for about 1% of all spinal tumors, predominantly in the thoracic spine (65%) [1]. Spontaneous arachnoid cysts are more common in males with a peak incidence in the second decade of life [2].

Theories about its pathogenesis are diverse, one of which suggests that it is due to a defect in the dura mater that gradually grows until it causes a leak with a unidirectional flow of cerebrospinal fluid (CSF). Therefore, it is proposed that the existence of a defect as a solution of continuity between the subarachnoid space and the cystic lesion allows the pressure difference to stimulate the flow of CSF in the direction of the subarachnoid space toward the cyst [3].

It has also been reported that a history of trauma, inflammation, or infections causes arachnoid adhesions; these adhesions may be related to the formation of cysts with a valve effect that facilitates herniation of the arachnoid membrane and the resulting accumulation of CSF. Theories about its origin converge on the existence of communication between the subarachnoid space, whether idiopathic (congenital) or acquired [4].

These lesions are generally asymptomatic; however, when there are clinical manifestations, it is due to a mass-occupying effect and its location. Clinical manifestations can vary from radicular pain, sensory symptoms, motor deficit, autonomic deficit, and even manifestations of myelopathic compression [5].

In this article, we describe the successful management of a compressive spinal epidural arachnoid cyst that was operated on using two multilevel laminectomies centered on both poles of the extensive lesion.

## Technical report

We report the case of a 33-year-old male patient with no past medical history presenting to our tertiary referral center. He presented to the outpatient clinic complaining of progressive gait disorder for two years, impacting his daily life to the point of inability to ambulate.

On physical examination, we found that the patient had paraparesis in the lower limbs, with 2/5 and 3/5 (Medical Research Council [MRC] scale) proximally and distally for the left and right limbs, respectively. Sensory deficit was observed from levels T8 with alteration in proprioception and hyposensitivity. Increased patellar and Achilles reflexes were found in the right leg, and continuous clonus is evident in the left ankle. Extensor plantar response (Babinski sign) was present bilaterally. Sphincter tones in the bladder and anus were preserved.

**Figure 1:**
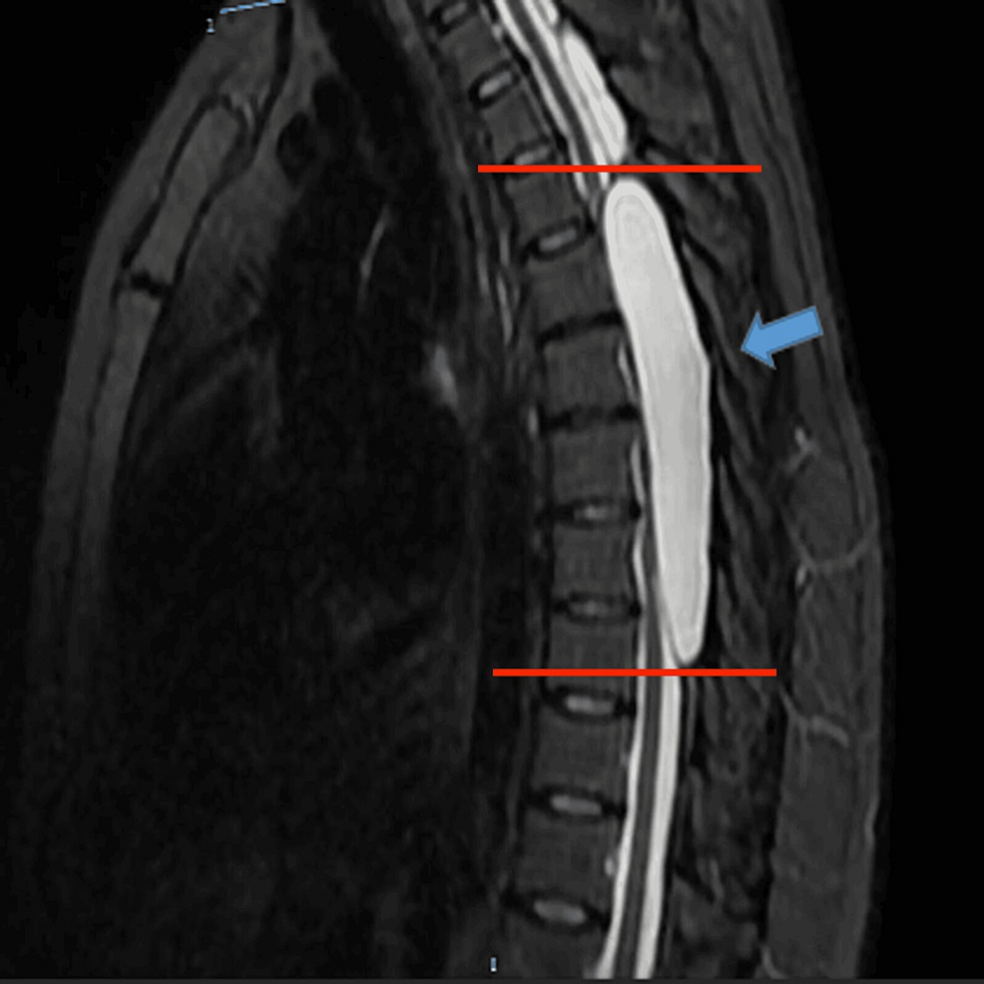
Preoperative imaging study The T2 MRI sequence sagittal section shows a hyperintense epidural lesion (a blue arrow) from level T3 to T8 (marked with red lines), compressing the spinal cord from dorsal to ventral with defined borders. MRI: Magnetic resonance imaging.

The imaging of the lesion is depicted in Figure 1. In the magnetic resonance study with CSF kinetics, no communication was observed between the epidural lesion and the subdural space. The analysis of the T1- and T2-weighted MRI determined the epidural cystic lesion. Therefore, an incomplete pyramidal syndrome ASIA C (American Spinal Injury Association) was integrated with pyramidal release secondary to an extensive thoracic spinal epidural cystic lesion.

Technical description of the surgical procedure

**Figure 2:**
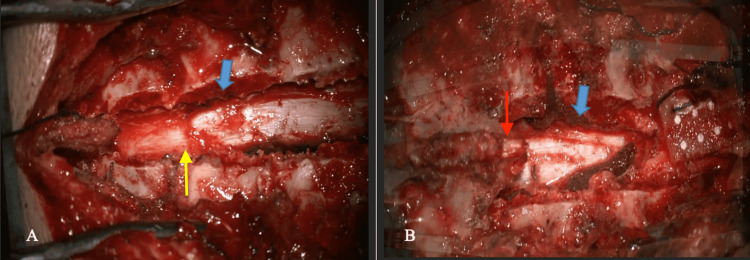
Surgical approach (A) Section of the interspinous and supraspinous ligaments through a posterior thoracic approach (blue arrows). The proximal pole of the exposed cyst and lesion-cord interface is indicated by the yellow arrow. (B) Distal pole of exposed cyst (red arrow).

With the patient in a prone position, a surgical procedure was performed by approaching through the midline, exposing the thoracic vertebral levels T3-T5 and T8-T9, corroborated with the use of an intraoperative fluoroscope, sectioning supraspinous and interspinous ligaments. Laminectomies with en bloc flavectomy were performed at the T3, T4, and T5 levels to expose the proximal pole of the cystic lesion. This same procedure was replicated at levels T8 and T9 to identify the distal pole (Figure 2).

During the intraoperative period, both macroscopic examination and the use of a surgical microscope revealed a united bilobulated cystic lesion in an epidural location.

**Figure 3:**
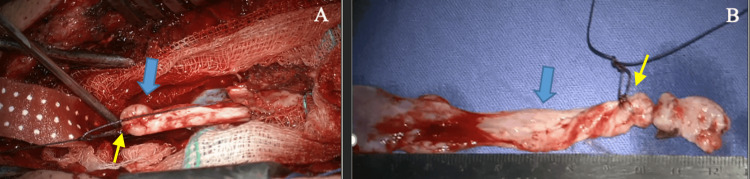
Cystic lesion (A) Gradual extraction of the cystic lesion (blue arrows). (B) Total extraction of the lesion, 12 cm length. The purse-shaped suture is indicated by the yellow arrows.

**Figure 4:**
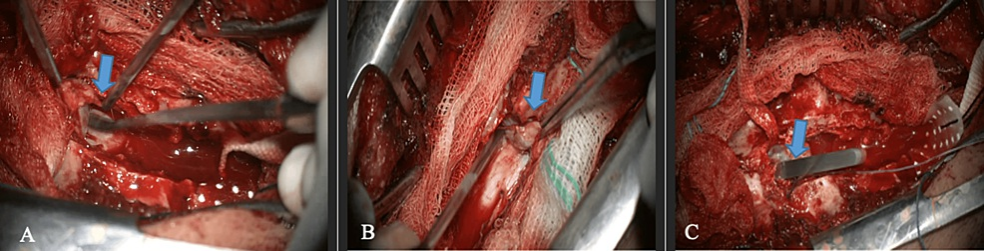
Folding maneuver (A) Dissection of the cystic lesion with Martel dissector (blue arrow). (B and C) The proximal pole suture and “folding maneuver” (blue arrows).

The cystic lesion was dissected from the bases of the spinous processes and laminae at levels T6 and T7, using a Martel dissector. Subsequently, a purse-shaped suture was placed in its proximal pole (Figure 3, Panel A), and then the “folding maneuver” was performed on the longitudinal axis of the cystic lesion, gently pulling toward the distal pole (Figure 4).

Subsequently, continuous traction of the cystic lesion was performed until the cystic lesion was completely removed. A sweep was performed on the dura mater without observing CSF leak. The actual length of the cystic lesion was 12 cm (Figure 3).

**Figure 5:**
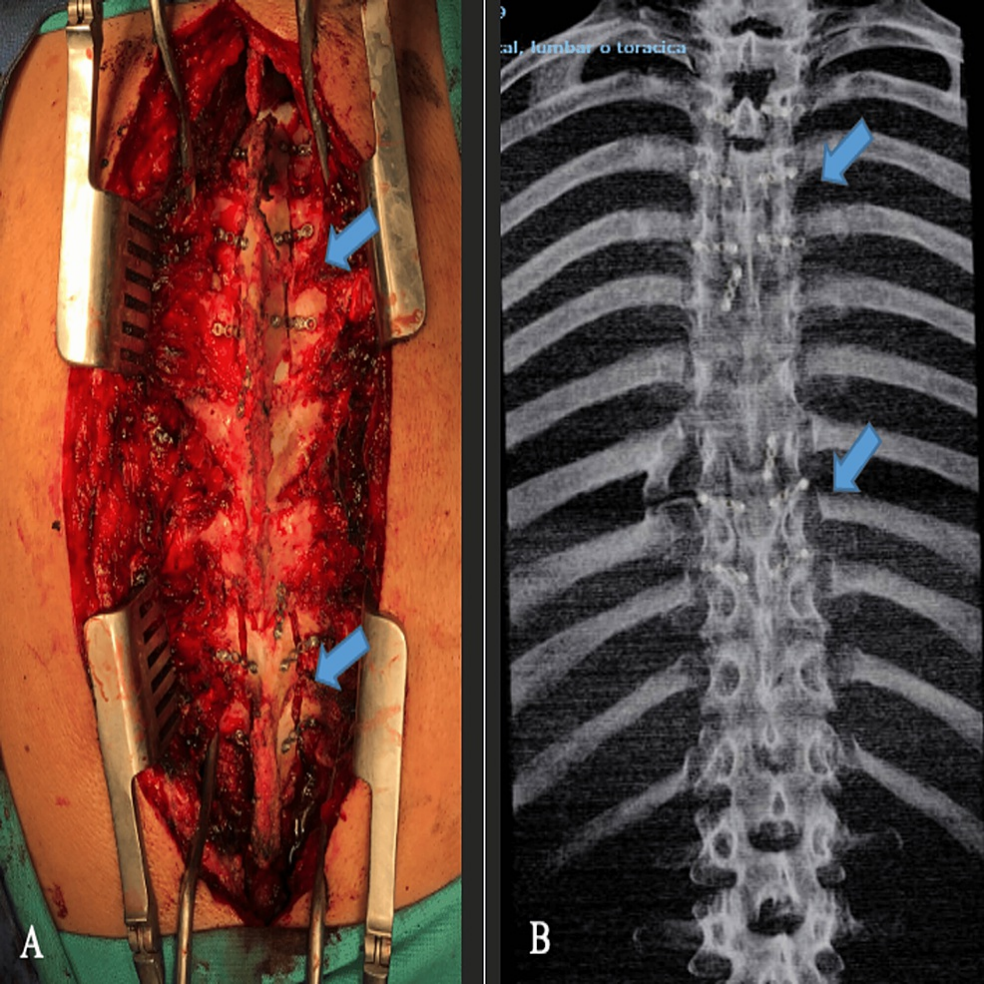
Reconstruction of the approach and postsurgical image (A) Laminoplasty with titanium plate and screws (blue arrows). (B) A simple coronal radiograph showing laminoplasties (blue arrows).

The reconstruction continued with laminoplasty for which titanium miniplates and screws were used (Figure 5), after verifying adequate hemostasis. As a result, the procedure ended without complications. The histopathological study was reported as compatible with arachnoid and dural tissue. There were no short- or long-term complications secondary to the surgical resection. In the follow-up during the outpatient consultation, the patient's muscle strength improved over the course of several months, and with physical rehabilitation, the patient was able to stand and walk slowly.

## Discussion

Spinal arachnoid cysts (SAC) are rare lesions, especially those with epidural and giant presentation, with extradural ones being less than 1% of these lesions. The etiology has not been elucidated due to its rarity; however, there is a valve effect mechanism with unidirectional CSF accumulation at a site of meningeal injury [1].

There are classifications of arachnoid cysts; however, little is described in the literature about dural cysts [6]. Three of these types of cysts are classified in Table 1.

**Table 1:** Classification of spinal meningeal cysts Source: Ref. [7].

Type	Description
I	Extradural meningeal cyst without nerve fibers
IA	Extradural arachnoid
IB	Sacral meningocele
II	Extradural meningeal cyst with nerve fibers (Tarlov cyst)
III	Intradural meningeal cyst

Conservative management with iterative clinical and radiological follow-up is generally preferred in the case of asymptomatic patients. However, surgery may be warranted to cope with symptomatic cases or rapidly evolving lesions.

Hamamcioglu et al. reported a giant lesion that was from C4 to T2; they proceeded with wide resection through total and extensive laminectomy of all segments for complete resection [8]. On the other hand, Lee et al. proposed a technique based on minimal hemilaminectomy for an extension cyst from T10 to L1 without complications, reducing the risk of xiphosis associated with a wide approach [3]. However, this lesion was shorter than previously reported.

As already mentioned, an extensive approach can be associated with greater complications such as hematoma, pain, and risk of postoperative xiphosis in up to 33%-100% of cases [9]. Therefore, it is imperative to implement an adequate laminoplasty in giant cysts and the least number of possible laminectomies. Our technique uses a minimal and necessary approach; therefore, instrumentation was not necessary for these injuries that do not disrupt the bone vertebral structure. Our technique has the potential advantage of limiting the risk of long-term complications associated with extended single laminectomy.

Fam et al. reported a series of 22 patients in 10 years with SAC; of these patients, 19 underwent surgery. However, only 13 underwent conventional laminoplasty, and the rest underwent pedicle ligation or fenestration/marsupialization, reporting complete resolution by MRI in 14 out of 16 patients [4].

Most of the reported series proposed conventional laminoplasties that are adapted to the length of the cyst, which is convenient in small lesions. However, in giant lesions like ours, great surgical advances are not reported. Furthermore, our proposed technique is pioneering and ideal in giant, longitudinal lesions in which there is no evidence of a dural lesion to repair along the fold path.

## Conclusions

Total removal of a giant symptomatic spinal epidural arachnoid cyst through a two-stage posterior laminoplasty seems to be a viable option. This surgical procedure successfully allowed the removal of the cyst entirely while mitigating complications as compared with a traditional extended single laminectomy.

In this study, the technique of en bloc and alternating laminectomies, plus folding of the cystic lesion, is proposed as a pioneering option for the resection of extensive epidural cysts. This approach provides sufficient exposure to the cystic lesion, avoiding laminectomies as extensive as those reported in the series for giant lesions and the risk of producing secondary xiphosis. A reduction in surgical time is assumed with a decrease in the probable volume of bleeding and postsurgical pain associated with an extensive approach. Further study have to be performed to confirm the safety of such surgical planning.

## References

[1] Tureyen K, Senol N, Sahin B, Karahan N (2009). Spinal extradural arachnoid cyst. Spine J.

[2] Kim KS, Weinberg PE (1986). Magnetic resonance imaging of a spinal extradural arachnoid cyst. Surg Neurol.

[3] Lee HJ, Cho WH, Han IH, Choi BK (2013). Large thoracolumbar extradural arachnoid cyst excised by minimal skipped hemilaminectomy: a case report. Korean J Spine.

[4] Fam MD, Woodroffe RW, Helland L, Noeller J, Dahdaleh NS, Menezes AH, Hitchon PW (2018). Spinal arachnoid cysts in adults: diagnosis and management. A single-center experience. J Neurosurg Spine.

[5] Bitaraf MA, Zeinalizadeh M, Meybodi AT, Meybodi KT, Habibi Z (2009). Multiple extradural spinal arachnoid cysts: a case report and review of the literature. Cases J.

[6] Chin Neurosurg Jl (2016). Classification, mechanism and surgical treatments for spinal canal cysts. Sun Chinese Neurosurgical Journal.

[7] Nabors MW, Pait TG, Byrd EB, Karim NO, Davis DO, Kobrine AI, Rizzoli HV (1988). Updated assessment and current classification of spinal meningeal cysts. J Neurosurg.

[8] Hamamcioglu MK, Kilincer C, Hicdonmez T, Simsek O, Birgili B, Cobanoglu S (2006). Giant cervicothoracic extradural arachnoid cyst: case report. Eur Spine J.

[9] Amhaz HH, Fox BD, Johnson KK, Whitehead WE, Curry DJ, Luerssen TG, Jea A (2009). Postlaminoplasty kyphotic deformity in the thoracic spine: case report and review of the literature. Pediatr Neurosurg.

